# Whole-Genome Sequence of Multidrug-Resistant *Campylobacter coli* Strain COL B1-266, Isolated from the Colombian Poultry Chain

**DOI:** 10.1128/genomeA.00130-16

**Published:** 2016-03-17

**Authors:** Johan F. Bernal, Pilar Donado-Godoy, Alejandra Arévalo, Carolina Duarte, María E. Realpe, Paula L. Díaz, Yolanda Gómez, Fernando Rodríguez, Richa Agarwala, David Landsman, Leonardo Mariño-Ramírez

**Affiliations:** a Corporación Colombiana de Investigación Agropecuaria (CORPOICA), Centro de investigación Tibaítata, Cundinamarca, Colombia; b Instituto Nacional de Salud, Grupo Microbiología, Bogotá, Colombia; c National Center for Biotechnology Information, National Library of Medicine, National Institutes of Health, Bethesda, Maryland, USA

## Abstract

*Campylobacter coli* is considered one of the main causes of food-borne illness worldwide. We report here the whole-genome sequence of multidrug-resistant *Campylobacter coli* strain COL B1-266, isolated from the Colombian poultry chain. The genome sequences encode genes for a variety of antimicrobial resistance genes, including aminoglycosides, β-lactams, lincosamides, fluoroquinolones, and tetracyclines.

## GENOME ANNOUNCEMENT

*Campylobacter* spp. are spiral-shaped bacteria capable of surviving in a wide range of environments, causing disease in humans and animals (1) and are also considered one of the main causes of food-borne illness worldwide (2, 3). In industrialized countries *Campylobacter coli* is the most common cause of community-acquired inflammatory enteritis (4). Other studies have suggested that the consumption of undercooked poultry and/or the inappropriate handling of raw poultry are risk factors for human campylobacteriosis (5, 6). Although most *Campylobacter* sp. infections are self-limiting, severe infections can occur and can even be fatal for very young children, the elderly, and immunosuppressed people (7). Some strains of *Campylobacter* spp. are increasingly resistant to most clinically important antibiotics, generating concern among public health authorities. The *Campylobacter* sp. described here is part of a comprehensive prevalence survey from the Colombian Integrated Program for Antimicrobial Resistance Survival (COIPARS), a national initiative to evaluate and to mitigate the spread of antimicrobial resistance associated to zoonotic pathogens in Colombia (8, 9). The isolate COL B1-266 is a multidrug-resistant member of a *Campylobacter coli* pulsed-field gel electrophoresis (PFGE) cluster composed of eight isolates from different stages of the poultry chain production (including drinking water at the farm, hatchery, and broiler farm and carcasses at slaughterhouses), suggesting dissemination through the poultry chain. This study was developed with the collaboration of two commercial producers in Colombia.

Here, we report the first whole-genome sequence of a Colombian multidrug-resistant strain, *Campylobacter coli* COL B1-266. Genomic DNA was isolated from an overnight culture using the PureLink Genomic DNA minikit (Invitrogen), and DNA libraries were prepared using the SureSelect QXT sample preparation kit (Agilent). The libraries were prepared according to the manufacturer’s instructions and sequenced on an Illumina HiScanSQ instrument with 2 × 151-bp paired-end reads, according to standard Illumina protocols. The genome was assembled using the reference-guided assembler ARGO, developed at NCBI, and the *de-novo* assembler SPAdes (10) to create initial assemblies of the reads. These were combined to produce the final assembly using a conservative process that incorporates pieces of *de-novo* assembly in the reference-guided assembly. The genome sequence of strain COL B1-266 consisted of 121 contigs, yielding total sequences of 1,948,500 bp. The overall G+C content of the isolate was determined to be 31%. The contigs were annotated using the NCBI Prokaryotic Genome Automatic Annotation Pipeline (PGAAP) and have been deposited at GenBank. The results of the genome annotation revealed 2,106 genes, 2,062 coding sequences, 120 pseudogenes, 3 rRNAs, 38 tRNAs, and 3 noncoding RNAs.

A search for resistance-associated genes present in the isolate was performed using ResFinder version 2.1 (11) and enriched using RAST version 2.0 (12) with default parameters. We found antimicrobial resistance genes for aminoglycosides (Aph 3′-III, Aad A2, and Sat1), β-lactams (blaOXA-61: beta-lactamase EC 3.5.2.6 or Cam-1), lincosamides (InuC), fluoroquinolones (gyrA and gyrB), tetracyclines (EF-G and TetO), and some genes associated with increased drug resistance (Efflux pumps: MFS, MacA, MacB, RND, and OM).

### Nucleotide sequence accession numbers.

This whole-genome shotgun project has been deposited in DDBJ/EMBL/GenBank under the accession number LKIV00000000. The version described in this paper is the second version. The BioProject accession is PRJNA296596.

## References

[1] Silva J, Leite D, Fernandes M, Mena C, Gibbs PA, Teixeira P 2011 *Campylobacter* spp. as a foodborne pathogen: a review. Front Microbiol 2:200. doi:10.3389/fmicb.2011.00200. 21991264 PMC3180643

[2] Scallan E, Hoekstra RM, Angulo FJ, Tauxe RV, Widdowson MA, Roy SL, Jones JL, Griffin PM 2011 Foodborne illness acquired in the United States—major pathogens. Emerg Infect Dis 17:7–15. doi:10.3201/eid1701.091101p1. 21192848 10.3201/eid1701.P11101 PMC3375761

[3] Williams MS, Golden NJ, Ebel ED, Crarey ET, Tate HP 2015 Temporal patterns of *Campylobacter* contamination on chicken and their relationship to campylobacteriosis cases in the United States. Int J Food Microbiol 208:114–121. doi:10.1016/j.ijfoodmicro.2015.05.018. 26065728

[4] Allos BM 2001 *Campylobacter jejuni* infections: update on emerging issues and trends. Clin Infect Dis 32:1201–1206. doi:10.1086/319760. 11283810

[5] Bearth A, Cousin M-E, Siegrist M 2014 Poultry consumers’ behaviour, risk perception and knowledge related to campylobacteriosis and domestic food safety. Food Control 44:166–176. doi:10.1016/j.foodcont.2014.03.055.

[6] Strachan NJ, Forbes KJ 2010 The growing UK epidemic of human campylobacteriosis. Lancet 376:665–667. doi:10.1016/S0140-6736(10)60708-8. 20663545

[7] Luangtongkum T, Jeon B, Han J, Plummer P, Logue CM, Zhang Q 2009 Antibiotic resistance in *Campylobacter*: emergence, transmission and persistence. Future Microbiol 4:189–200. doi:10.2217/17460913.4.2.189. 19257846 PMC2691575

[8] Donado-Godoy P, Castellanos R, León M, Arevalo A, Clavijo V, Bernal J, León D, Tafur MA, Byrne BA, Smith WA, Perez-Gutierrez E 2015 The establishment of the Colombian Integrated Program for Antimicrobial Resistance Surveillance (COIPARS): a pilot project on poultry farms, slaughterhouses and retail market. Zoonoses Public Health 62(suppl 1):58–69. doi:10.1111/zph.12192. 25903494

[9] Donado-Godoy P, Bernal JF, Rodríguez F, Gomez Y, Agarwala R, Landsman D, Mariño-Ramírez L 2015 Genome sequences of multidrug-resistant *Salmonella enterica* serovar paratyphi B (dT+) and Heidelberg strains from the Colombian poultry chain. Genome Announc 3(5):e01265-15. doi:10.1128/genomeA.01265-15. 26494672 PMC4616196

[10] Bankevich A, Nurk S, Antipov D, Gurevich AA, Dvorkin M, Kulikov AS, Lesin VM, Nikolenko SI, Pham S, Prjibelski AD, Pyshkin AV, Sirotkin AV, Vyahhi N, Tesler G, Alekseyev MA, Pevzner PA 2012 SPAdes: a new genome assembly algorithm and its applications to single-cell sequencing. J Comput Biol 19:455–477. doi:10.1089/cmb.2012.0021. 22506599 PMC3342519

[11] Zankari E, Hasman H, Cosentino S, Vestergaard M, Rasmussen S, Lund O, Aarestrup FM, Larsen MV 2012 Identification of acquired antimicrobial resistance genes. J Antimicrob Chemother 67:2640–2644. doi:10.1093/jac/dks261. 22782487 10.1093/jac/dks261 PMC3468078

[12] Aziz RK, Bartels D, Best AA, DeJongh M, Disz T, Edwards RA, Formsma K, Gerdes S, Glass EM, Kubal M, Meyer F, Olsen GJ, Olson R, Osterman AL, Overbeek RA, McNeil LK, Paarmann D, Paczian T, Parrello B, Pusch GD, Reich C, Stevens R, Vassieva O, Vonstein V, Wilke A, Zagnitko O 2008 The RAST server: rapid annotations using subsystems technology. BMC Genomics 9:75. doi:10.1186/1471-2164-9-75. 18261238 PMC2265698

